# Detection of Voltage Anomalies in Spacecraft Storage Batteries Based on a Deep Belief Network

**DOI:** 10.3390/s19214702

**Published:** 2019-10-29

**Authors:** Xunjia Li, Tao Zhang, Yajie Liu

**Affiliations:** College of Systems Engineering, National University of Defense Technology, Changsha 410073, China; lixunjia13@nudt.edu.cn (X.L.); zhangtao@nudt.edu.cn (T.Z.)

**Keywords:** battery, spacecraft, telemetry data, performance degradation, deep belief network, anomaly detection

## Abstract

For a spacecraft, its power system is vital to its normal operation and capacity to complete flight missions. The storage battery is an essential component of a power system. As a spacecraft spends more time in orbit and its storage battery undergoes charge/discharge cycles, the performance of its storage battery will gradually decline, resulting in abnormal multivariate correlations between the various parameters of the storage battery system. When these anomalies reach a certain level, battery failure will occur. Therefore, the detection of spacecraft storage battery anomalies in a timely and accurate fashion is of great importance to the in-orbit operation, maintenance and management of a spacecraft. Thus, in this study, based on storage battery-related telemetry parameter data (including charge/discharge currents, voltages, temperatures and times) downloaded from an in-orbit satellite, a voltage anomaly detection algorithm for spacecraft storage batteries based on a deep belief network (DBN) is proposed. By establishing a neural network (NN) model depicting the correlations between each of the variables of temperature, current, pressure and charge/discharge times and voltage, this algorithm supports the detection of anomalies in the state-of-health of a storage battery in a timely fashion. The proposed algorithm is subsequently applied to the storage battery of the aforementioned in-orbit satellite. The results show the following. The anomalies detected using the proposed algorithm are more reliable, effective and visual than those obtained using the conventional multivariate anomaly detection algorithms. Compared to the classic backpropagation NN-based algorithm, the DBN-based algorithm is notably advantageous in terms of the model training time and convergence.

## 1. Introduction

Spacecraft telemetry data are real-time data acquired by sensors and transmitted to telemetry terminals via telemetry links; they are also the only data based on which ground management personnel determine and evaluate the working conditions and state-of-health (SoH) of a spacecraft. As the structure and function of spacecraft systems become increasingly complex, telemetry data-based system checks and state evaluations are becoming increasingly important. Storage batteries are important spacecraft components. As a spacecraft spends more time in orbit, its storage battery will experience a series of faults, such as performance degradation and output power decreases. In severe cases, these faults will render the power system unable to provide a normal power supply to relevant loads, thereby affecting the operation of the spacecraft. A statistical analysis of the collected data regarding 324 faults experienced by 227 satellites owned by countries outside China in the period from 1994–2014 found that 84 were direct power system faults, which account for 26% of the faults [1]. Therefore, to improve the safety and reliability of spacecraft systems, it is necessary to make full use of collected telemetry data to detect spacecraft storage battery anomalies in a timely and accurate fashion, thereby providing effective support for fault diagnosis and preventing detrimental disasters to spacecraft operation.

Common storage battery faults include performance degradation, short- and open-circuit failures of single battery cells and failures caused by charge/discharge circuit faults [1]. Amid this background, in this study, based on the orbital characteristics of a certain satellite, the collected telemetry data for its storage battery and the needs for detecting anomalies in its SoH, a voltage anomaly detection algorithm for spacecraft storage batteries based on a deep belief network (DBN) is proposed.

## 2. Related Works

In view of the important research and application value of anomaly detection in such fields as health management of important equipment (e.g., spacecraft, missiles and numerically controlled machine tools), researchers have proposed various anomaly detection algorithms. Based on the working principle, overall, these algorithms can be categorized into six types [2,3,4], namely, threshold-based, statistical analysis-based, nearest neighbour-based, cluster analysis-based, classification-based and prediction-based algorithms. Each type of algorithm has its own attributes and application scenarios. Threshold-based algorithms are simple and fast and consume almost no computational resources. However, on one hand, the threshold range is difficult to set due to its relationship with the working mode; on the other hand, it is impossible to identify anomalies within the threshold [5]. Compared to threshold-based algorithms, statistical analysis-based algorithms have higher learning abilities and relatively wider ranges of applications. These algorithms each establish a model based on known normal data and use the model to detect anomalies in test data. However, the models established by these algorithms based on datasets with specific distribution patterns lack universality and need to be updated in a timely fashion as the system changes to ensure high detection accuracy [6,7]. Nearest neighbour-based algorithms classify abnormal data by measuring the similarity index. There are two similarity indices, namely, density- and distance-based similarity indices, that follow the assumption that normal data appear in dense areas due to their high degree of similarity, whereas abnormal data are distant from normal data due to their low degree of similarity to most normal data [8]. Cluster analysis-based algorithms obtain clustering results by unsupervised learning and analyse data to determine whether they are within normal clusters to detect anomalies [9]. The precondition for using the aforementioned two types of algorithms is that the majority of the data samples are normal data and that only a small proportion are abnormal data. Additionally, the anomalies detected by these algorithms are very sensitive to distance functions and noise, and these algorithms also overlook the correlations between data. Classification-based algorithms conduct supervised learning by marking the data with normal and abnormal labels [10,11]. However, data labelling, in many cases, requires specialized personnel, and this process is overly complicated and costly, which will result in reduced detection efficiency and accuracy when detecting anomalies in massive amounts of data. In particular, it is worth mentioning that under practical conditions, spacecraft telemetry parameters often reach ground stations via measurement and control channels in the form of streaming data and, consequently, the aforementioned statistical analysis-, cluster analysis- and classification-based detection algorithms are unable to meet the anomaly detection requirements. In contrast, prediction-based algorithms primarily detect anomalies by comparing the measured data with the output data predicted by models. Prediction-based algorithms rely on pre-trained models, require no a priori knowledge, are independent of labels, and can detect anomalies in real time [12,13]; as a result, they are more applicable to anomaly detection compared to the aforementioned algorithms. Hence, a prediction-based algorithm is employed in this study to detect abnormal storage battery conditions.

As mentioned previously, spacecraft storage battery degradation is a typical of multivariate anomaly type. Considering that spacecraft systems are complex and often consist of multiple subsystems, their telemetry data are characterized by high volumes, high dimensionality, complex parameter relationships and the presence of noise disturbance. As a result, multivariate anomalies are more difficult to detect than conventional anomalies. Therefore, to detect anomalies in multivariate spacecraft telemetry data, it is often necessary to reduce their dimensionality and extract their features. On this basis, a statistical analysis- or classification-based algorithm is also required. Based on the working principle, currently, relevant algorithms for detecting multivariate anomalies in telemetry data are primarily categorized into four types, namely, subspace calculation-based, basic model error reconstruction-based, generative model estimation-based and structural graph-based algorithms [14].

Of the aforementioned four types of algorithms, the subspace calculation- and basic model error reconstruction-based algorithms are the ones most commonly used. A subspace calculation-based algorithm treats a multivariate time series as multiple independent samples distributed in a high-dimensional space. Based on a certain mapping rule, data samples can be embedded into low-dimensional spaces [15,16]. Thus, anomalies can be detected based on the degree of deviation of the samples in the subspaces. A basic model error reconstruction-based algorithm preserves the relationships and time-domain correlations between the variables in the original multivariate time series in the processed samples relatively well using sparse coding and dimensionality-reduction techniques [17,18]. The idea of this type of algorithm is described as follows. For a normal telemetry dataset $D_{train}=\{y_{1},\ldots ,y_{N}\}$, a pair of projections, f ($f:y_{i}\in R^{D}\to z_{i}\in R^{d}(D>d)$ is a “reduced-dimensional” projection from the high-dimensional space to a low-dimensional space) and g ($g:z_{i}\in R^{d}\to y_{i}\in R^{D}$ is a “reconstructed” projection from a low-dimensional space to the high-dimensional space), is learned at the training stage. Based on this pair of projections (*f* and *g*), each reconstructed output ${\hat{y}}_{i}=g(f(y_{i}))(i=1,\ldots ,N)$ is allowed to approximate the original data sample *y_i_*. The specific forms of *f* and *g* vary between algorithms [19]. When anomalies occur, because abnormal patterns are not included in any of the patterns of the training data, they will not be reconstructed by *f* and *g*. These anomalies are reflected by two statistics, namely, the squared prediction error (*SPE*) statistic and the Hotelling $T^{2}$ statistic. The *SPE* statistic is mainly used to calculate the total reconstruction error for each sample. The Hotelling $T^{2}$ statistic is predominantly used to describe the degree of variation of a sample within the model space. The higher the values of the statistics are, the higher the abnormality degree is. Generally, a decision control limit is established based on the statistical characteristics of the normal sample data for each of these two metrics. A test sample is considered an anomaly if its statistical metrics exceed the control limits. Regarding specific research, Yairi et al. [19] applied an anomaly dimensionality reduction-reconstruction framework to spacecraft systems and compared several representative linear and non-linear dimensionality reduction algorithms, including principal component analysis (PCA), kernel PCA and the Gaussian process latent variable model. Cheng et al. [20] and Miao et al. [21] used the locally linear embedding (LLE) algorithm to ameliorate the problem of the conventional linear dimensionality reduction algorithms—they are unable to relatively satisfactorily describe the actual data structures in subspaces. Additionally, they also improved the LLE algorithm and used it to detect anomalies in spacecraft systems. For this type of algorithm, the key lies in how to determine the dimensionality after the dimensionality reduction. If a proper dimensionality is selected, anomalies can be relatively accurately detected. Moreover, the detection efficiency also varies between algorithms.

The generative model estimation-based algorithms identify data points with low probabilities as abnormal points by establishing a generative model for the data. These algorithms have a solid basis in mathematical theory and are able to effectively reflect spatial and temporal relations in a time series if the estimates are accurate. Commonly used models include the vector autoregressive and state space models. Li et al. [22] used the inner product of vectors in the principal directions as the metric for measuring anomalies. Additionally, they also characterized the distribution of vectors in the principal directions based on the von Mises–Fisher distribution to detect anomalies in multivariate time series. Moreover, they estimated the parameters of the distribution model by training on historical data. Furthermore, they determined the occurrence of anomalies by calculating the probabilities of vectors of test data in the principal directions in the training model. This algorithm has several disadvantages. Specifically, it is unable to accurately estimate the data distribution and model parameters when there is no available a priori knowledge. Additionally, there are complex relationships between the telemetry data parameters, and it is therefore difficult to establish an accurate model. This leads to reduced detection accuracy.

Structure graph-based algorithms detect anomalies by establishing a graph structure for the properties in a time series and are able to simultaneously take into consideration the spatial and time-domain correlations between data. Idé et al. [23] defined a neighbourhood graph based on the correlation between sensor signals under the assumption that under normal circumstances, the graph structure, to a certain extent, remains stable and is unaffected by fluctuations in the experimental conditions. Qiu et al. [24] proposed to use Granger graphical models as an anomaly-detection approach. They concluded that this approach is efficient in detecting anomalies, and its results are easy to explain. Cheng et al. [25] proposed an anomaly-detection algorithm that involves the construction of a kernel matrix and its aligned graph structure. However, if a relatively large number of variables are involved, the graph will have a large number of edges, resulting in high algorithm complexity. In this situation, it is necessary to employ certain techniques to reduce the computational complexity of the algorithm.

The aforementioned classical multivariate anomaly-detection algorithms are unsuitable for spacecraft storage battery degradation. For dimensionality reduction and reconstruction algorithms, because SPE and $T^{2}$ are distance-based statistics, massive volumes of high-dimensional telemetry data will increase the complexity of their calculations. Additionally, telemetry data do not completely obey a Gaussian distribution and often exhibit multimodal characteristics. Therefore, the decision control limits for these two metrics are unable to closely contain all the normal data ranges. This will lead to relatively high omission rates. Moreover, the Z-score method is often used in data pre-processing. As a result, it is impossible to accurately describe actual global data conditions. For generative model-based algorithms, because a spacecraft storage battery system has a large number of parameters with complex interactions, it is difficult to establish an accurate model. This results in low accuracy. Regarding the structure graph-based algorithms, due to the stability of a graph structure, it is unable to characterize the evolutionary characteristics of a spacecraft system. Additionally, as a result of the large number of parameters, the graph will have a large number of edges, resulting in high complexity.

Apart from the detection of spacecraft storage battery anomalies, relevant research has also studied the detection of anomalies in storage batteries in such objects as electric vehicles. This type of research shares the following common features. The detected anomalies are verified by static battery experiments under strict conditions. Additionally, the parameters are measured using high-precision sensors. These parameters include voltage, current, state-of-charge (SoC), temperature, capacity, internal resistance, number of cycles and time. Zhao et al. [26] measured the parameters of a battery system (including the voltage, current, SoC and temperatures at the characteristic points of the battery pack) and detected anomalies using the local outlier factor algorithm in conjunction with the diagnostic algorithm for cluster outliers. Mingant et al. [27] proposed a diagnostic technique based on the analysis of free voltage and current signals to provide a so-called “quasi-electrochemical impedance spectrum”. Additionally, they also diagnosed the SoH of lithium (Li)-ion batteries based on parameters such as the battery capacity and internal resistance. Liu and He [28] proposed a sensor fault detection and isolation scheme using an adaptive extended Kalman filter. Piao et al. [29] proposed an outlier detection algorithm for evaluating battery system safety. Nuhic et al. [30] diagnosed and predicted the battery health using a data-driven approach in combination with a support vector machine based on battery parameters such as SoC, capacity, number of cycles, temperature and time. Chen et al. [31] proposed a fault diagnosis algorithm based on a two-layer model and used it to examine the characteristics of external short-circuit faults in Li-ion batteries. Additionally, they performed an experiment on 10 Li-ion batteries with different SoCs. Dey et al. [32] proposed a two-state thermal model for thermal faults in Li-ion batteries. This model describes the dynamics of the surface and the dynamic changes in the core temperature of a battery cell. Additionally, this model takes into account the uncertainties in internal resistance, capacity and thermal parameters. Sidhu et al. [33] proposed an adaptive fault diagnosis approach based on a combination of multiple non-linear models. In this approach, the non-linear models represent signature faults. The impedance spectroscopy analysis of such parameters as the bulk resistance, charge-transfer resistance and double-layer resistance is combined with the equivalent circuit methodology. Extended Kalman filters are used to estimate the terminal voltages and generate residual signals, which are then used for fault diagnosis. Yan et al. [34] proposed a Lebesgue sampling-based fault diagnosis and prognosis framework for Li-ion batteries. The idea of this framework is to reduce the computational resources by an “execution only when necessary” or “execution based on needs” strategy. This approach was demonstrated to be effective in detecting reductions in the storage battery capacity. Lin et al. [35] proposed a SoC estimation approach based on an integration of multiple equivalent circuit models. The parameters (e.g., resistance and capacity) of each model are set using a genetic algorithm. Most of the aforementioned studies were conducted under strict and precise experimental conditions. For instance, in the study by Mingant et al. [27] and the study by Liu et al. [28], the experiments were conducted at room-temperature (25 ℃). Additionally, in the study by Liu et al. [28], the data sampling frequency was set to 10 Hz. Spacecraft operate in the complex space environment. On one hand, the environmental temperatures at which spacecraft operate can hardly be kept constant. In the aforementioned studies, some of the telemetry parameters of spacecraft storage batteries, such as the SoC, capacity and internal resistance, are often not provided. On the other hand, because spacecraft are affected by such factors as the memory size and measurement and control channel bandwidth, the sampling frequencies for their telemetry parameters are often low. Therefore, the aforementioned approaches cannot be directly applied to the detection of anomalies in spacecraft storage batteries.

In this study, based on the practical needs of spacecraft storage battery anomaly detection in combination with the characteristics of spacecraft telemetry data (e.g., high dimensionality, high volume and complex relationships), the deep-learning approach is introduced into storage battery anomaly detection. A DBN is used to establish a neural network (NN) model depicting the correlations of multiple parameters (e.g., temperature, current and charge/discharge times) with voltage. When the storage battery voltage starts declining, there will be multivariate correlations in the model. By detecting these multivariate anomalies, degradation or faults can be detected in a timely fashion before a storage battery fails.

Compared to the above studies, the main contributions of this study can be summarized in the following three aspects:

(1) Based on the characteristics of telemetry data for spacecraft storage batteries (e.g., high dimensionality and complex relations), and in view of the practical needs for online storage battery anomaly detection and the evolutionary pattern of spacecraft storage battery systems, a system state anomaly detection algorithm based on the internal correlations between multiple storage battery parameters is proposed.

(2) The deep-learning approach is introduced into storage battery anomaly detection. A DBN is used to establish an NN model depicting the correlations of multiple parameters (e.g., temperature, current and charge/discharge times) with voltage. When there is a significant decline in the SoH of a storage battery, there will be anomalies in the multivariate correlations in the model. By detecting these multivariate anomalies, degradation or faults can be detected in a timely fashion before the storage battery fails. Compared with the classic backpropagation NN-based algorithm, the DBN-based algorithm is notably advantageous in terms of its model training time and convergence.

(3) The algorithm has been utilized to detect the anomalies in the state-of-health of an in-orbit satellite’s storage battery, and the results show that this algorithm outperforms the conventional multivariate anomaly detection algorithms in terms of reliability, effectiveness and visibility.

## 3. Process of the Anomaly-Detection Algorithm

### 3.1. Parameter Extraction

First, the storage battery of the satellite, i.e., satellite X, examined in this study is introduced. Owing to the uniqueness of its orbit, the satellite X remains in the sunlit region for a relatively long period of time each year. In normal circumstances, when the satellite X operates in the sunlit region, the solar panels directly supply power to its loads, and its storage battery does not work and is only being charged by a weak current. Under this condition, the discharge current of the storage battery is basically zero, and the voltage of the storage battery remains basically unchanged. The solar panels and the storage battery supply power simultaneously only when there are enormous satellite loads. Under this condition, the voltage of the storage battery will decrease, and a charge current is generated. In less than one-third of the time each year, the satellite will frequently enter and exit the Earth’s shadow region. After entering the Earth’s shadow region, the whole satellite is powered by its storage battery. During this time, the voltage of the storage battery will continuously decrease, and the charge current remains zero during the discharge process. After the satellite re-enters the sunlit region, the solar array of the satellite starts charging the storage battery again, and the voltage of the storage battery also gradually increases. This process repeats continuously.

The telemetry data for spacecraft storage batteries to which ground personnel have access to include the voltages, currents, discharge/charge times, and temperatures. However, ground personnel are unable to obtain battery capacity and resistance, which are parameters commonly used in the ground performance evaluation and degradation testing of storage batteries.

**Figure 1:**
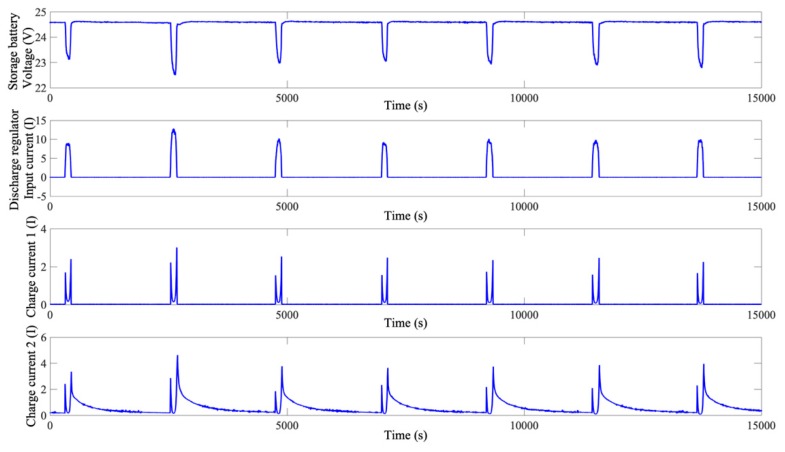
Relationships between the voltage of the storage battery and its current parameters.

The parameter extraction process is described as follows. First, the voltage of a storage battery is strongly correlated with such parameters as the charge/discharge currents, times and temperatures based on its working principle. Therefore, from the telemetry parameters, the aforementioned parameters are selected and recorded. Due to its orbital conditions, the spacecraft examined in this study will operate continuously in the sunlit region for a long period of time. During this process, the discharge current of its storage battery will basically remain zero, and its charge current is very small. Additionally, the voltage of the storage battery will remain basically unchanged. Therefore, the parameters of the storage battery when the satellite is in the sunlit region are meaningless and thus not selected. After the satellite enters the Earth’s shadow region, its storage battery will undergo charge/discharge cycles. Thus, the storage battery parameters in this process are extracted. Figure 1 shows the relationships between the parameters in the charge/discharge cycles after the satellite enters the Earth’s shadow region.

**Figure 2:**
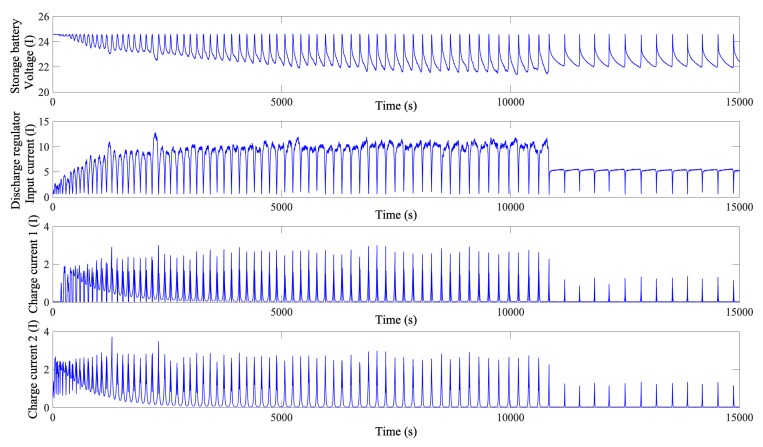
Relationships between the voltage of the storage battery and current parameters after the extraction process. (Subfigure 1 is the storage battery voltage, subfigure 2 is the discharge regulator input current, subfigure 3 is the charge current 1, subfigure 4 is the charge current 2.)

As demonstrated in Figure 1, the voltage of the storage battery first decreases and then increases in the discharge/charge process. This process is strongly correlated with each current parameter. However, after a full charge, the voltage of the storage battery reaches a stable value and remains basically unchanged for a long period of time. In this process, the current parameters become zero or remain at low levels. Thus, inputting the value of each parameter in this process into the NN will increase its structural complexity and significantly increase its training time. Additionally, the data for the period when the storage battery of the satellite is uncharged/discharged cannot directly facilitate the detection of satellite storage battery degradation. Thus, the extracted storage battery data for the charge/discharge stages are further screened. Figure 2 shows the relationship between the voltage and each current parameter after the extraction process. As demonstrated in Figure 2, the data for the period when the storage battery of the satellite does not undergo charge/discharge cycles are removed. As a result, the data volume is reduced considerably, which is favourable to the subsequent NN structure determination and large-scale NN training.

### 3.2. Neural Network (NN) Model Determination

#### 3.2.1. Introduction to Deep Belief Network (DBN)

According to the research status at home and abroad, the most widely used model in the multi-parameter correlation training method is the back-propagation neural network (BPNN) method [36], which is the most typical and currently the most widely used artificial neural network. The basic principle of the BP (back-propagation) neural network is to adjust and update the network parameters by calculating the error between the actual output value of the network and the expected output value, so that the error converges to a minimum. Its algorithm flow mainly includes two processes of forward propagation of information and backward propagation of error. In the case of forward propagation, the input information is transmitted from the input layer to the hidden layer, and is processed layer by layer by the hidden unit layer, and then acts on the output node. During this period, non-linear transformation is performed to generate the output information. If the actual output does not match the expected output, then the error back propagation process begins. During forward propagation, the state of each layer of neurons affects only the state of the next layer of neurons. Back propagation is to reverse the output error along the original connection path to the input layer through the hidden layer, and distribute the error to all the units in each layer. The error signal obtained from each layer is used as the basis for adjusting the weight of each unit. By adjusting the connection weights and thresholds between the layers, the error is reduced along the gradient direction. After repeated learning training, the network parameters (connection weights and thresholds) corresponding to the minimum error are determined, and the training stops. At this time, the trained neural network can process the non-linear conversion information with the smallest output error for the input information of similar samples. The BP neural network learning algorithm usually uses a gradient descent algorithm.

The BP neural network algorithm is relatively mature in terms of network theory and performance. Its outstanding advantage is that it has a strong non-linear mapping capability and a flexible network structure. The number of middle layers of the network and the number of neurons in each layer can be arbitrarily set according to specific conditions, and the performance differs depending on the structure. However, BP neural network also has some major defects, as follows: slow learning, even simple problems, generally require a great deal of iterative learning to converge; easy to fall into local minimum values and limited network promotion capabilities.

In order to overcome the above defects, this paper uses the deep belief network (DBN) as a network model for anomaly detection. Compared with the general generalized neural networks, deep learning methods can learn the characteristic information inherent in data. The related literature has proven that deep learning methods have better predictive effects [37,38]. The DBN is a typical deep-learning model [39].

**Figure 3:**
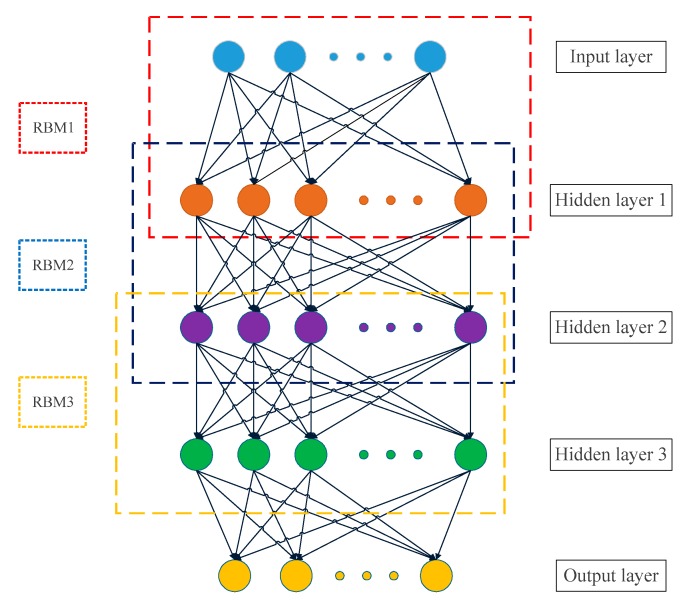
Schematic diagram of the deep belief network (DBN).

The deep belief network [39] is a deep neural network whose network structure is special in that it consists of a plurality of restricted Boltzmann machine (RBM) stacks and a layer of neural networks. The structure diagram of the DBN is shown in Figure 3. The training process of the DBN consists of two processes: pre-training and fine-tuning. The former can provide better initial values for the parameters of the network, while the latter can quickly search for the global optimal solution of the network parameters.

#### 3.2.2. Principle of the Restricted Boltzmann Machine (RBM)

The Restricted Boltzmann Machine [40] was originally invented by Hinton and Sejnowski in 1985, and until Hinton proposed a fast learning algorithm in 2005, it attracted the attention of experts and scholars. The RBM is a generated stochastic neural network that can learn the probability distribution of input data. The so-called restricted means that the complete map of the Boltzmann machine is limited to a bipartite graph, which can be regarded as a special topology of the Boltzmann machine. Therefore, the model is a random binary structure consisting of a visible layer that accepts input data and an hidden layer that outputs training results. In an RBM, there are connections between all visible nodes (i.e., neurons) and hidden nodes, but there are no connections between the visible nodes or between the hidden nodes, that is to say, the nodes are fully connected between layers but have no connection inside the layers (in the Boltzmann machine model, the nodes are fully connected between layers and inside each layer). These connections are bidirectional and symmetrical. Among them, each node (whether it is a hidden node or a visible node) has two states, as follows: the value is 1 when it is active and 0 when it is not activated. The meaning of the 0 and 1 states here is that the nodes that the model will select are used, the nodes that are active are used, and the nodes that are not active are not used. The activation probability of a node is calculated by the distribution function of the visible layer and the hidden layer nodes.

**Figure 4:**
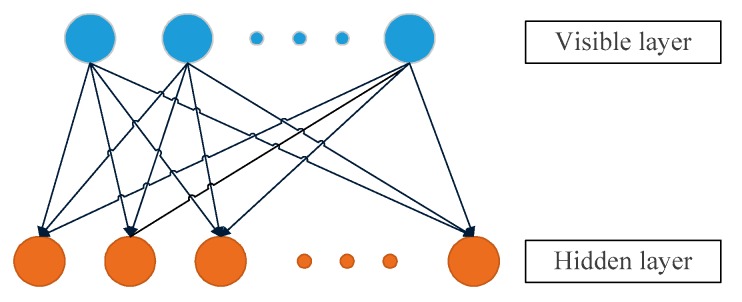
Schematic diagram of DBN.

The limitation of RBM makes it a more efficient training algorithm than the general Boltzmann machine, and the restricted Boltzmann machine is often used for different types of data generation models. Of course, its most important role is to serve as a learning module that forms a deep belief network. Figure 4 shows a schematic diagram of the structure of a single RBM.

The RBM is a model based on the energy function. The joint configuration energy of the visible layer and the hidden layer is expressed as follows:

(1) $$E\left(v,h;\theta \right)=-\sum_{ij}w_{ij}v_{i}h_{j}-\sum_{i}b_{i}v_{i}-\sum_{j}a_{j}h_{j},$$

where $v_{i}$ and $h_{j}$ represent the states of the visible node i and the hidden node j respectively. The states of the visible and hidden nodes are binary, that is, $v_{i}$ and $h_{j}\in \left\{0,1\right\}$. $w_{ij}$ represents the connection weight between the visible node i and the implicit node j. $a_{j}$ and $b_{i}$ represent the thresholds of the hidden node j and the visible node i respectively, and θ represents the model parameter set: θ={w,a,b}. The energy function is used to calculate the probability distribution of each pair of visible and implicit nodes. The lower the energy, the closer the network is to the desired target. After calculation, the probability distribution between the visible layer and the hidden layer is defined as follows:

(2) $$p\left(v,h\right)=\frac{1}{Z}e^{-E\left(v,h\right)},$$

where Z is a scoring function that represents the sum of the energy functions $e^{-E\left(v,h\right)}$ for all possible states and is used as a normalization factor, as follows:

(3) $$Z=\sum_{v,h}e^{-E\left(v,h\right)}.$$

Due to the special structural properties of the full connection between layers and no connection inside the layers of RBM model, the node states in the same layer are independent of each other, that is, when a visible cell state is given, the active states of the hidden cells are conditionally independent. Accordingly, when a hidden cell state is given, the activation probabilities of the visible cells are also conditionally independent.

Therefore, given the activation state of the visible node $v_{i}$, the probability that the state of the hidden it node $h_{j}$ is 1 is as follows:

(4) $$p_{h_{j}}=p\left(h_{j}=1|v\right)=\sigma \left(b_{i}+\sum_{i}w_{ij}v_{i}\right),$$

Correspondingly, after the state of the implicit node is given, the visible node $v_{i}{}^{\prime }$ is reconstructed, and the probability of its state being 1 is expressed as follows:

(5) $$p_{v_{i}{}^{\prime }}=p\left(v_{i}{}^{\prime }=1|h\right)=\sigma \left(a_{j}+\sum_{j}w_{ij}h_{j}\right).$$

Here σ(⋅) refers to the sigmoid activation function, as follows:

(6) $$\sigma \left(x\right)=\frac{1}{1+\exp\left(-x\right)},$$

#### 3.2.3. DBN Training Process

Pre-training process: The pre-training process is a process of initializing the connection weights and thresholds between the input layer and the hidden layer and between the hidden layers to obtain a better local optimal solution or even a global optimal solution. This process is implemented by an unsupervised greedy optimization algorithm for multiple restricted Boltzmann machines.

The steps of the restricted Boltzmann machine training process are as follows: first, select a set of training sample inputs to the visible node to obtain $v_{i}$; secondly, sample according to the probability $p\left(h_{j}=1|v\right)$ to obtain the state $h_{j}$ of the hidden node; then, according to the probability $p\left(v_{i}{}^{\prime }=1|h\right)$, the state $v_{i}{}^{\prime }$ of the visible node is reconstructed; finally, the error between the reconstructed visible node state $v_{i}{}^{\prime }$ and the true value $v_{i}$ of the visible node is calculated, if the error is too large, the state $h_{j}{}^{\prime }$ of the hidden node is reconstructed again according to the probability $p\left(h_{j}=1|v^{\prime }\right)$.

This process is repeated iteratively until the error between the reconstructed value of the visible node and the true value reaches a certain precision. The relevant parameters are also obtained according to the error calculation gradient, and the update formula is as follows:

(7) $$\Delta w_{ij}=\eta \left(\langle v_{i}h_{j}\rangle -\langle {v^{\prime }}_{i}{h^{\prime }}_{j}\rangle \right),$$

(8) $$\Delta a_{i}=\eta \left(\langle v_{i}\rangle -\langle {v^{\prime }}_{i}\rangle \right),$$

(9) $$\Delta b_{j}=\eta \left(\langle h_{j}\rangle -\langle {h^{\prime }}_{j}\rangle \right),$$

where η is the learning rate and 〈⋅〉 is the expected value used to calculate the corresponding data. The Monte Carlo sampling method is used to estimate the expected value in the formula. However, the conventional Monte Carlo method is very slow to calculate. The biggest reason is that it requires many step state transitions to ensure that the collected samples meet the target distribution. In 2002, Professor Hinton proposed the contrastive divergence (CD) method [41] for training restricted Boltzmann machines. At present, the contrastive divergence method has become the standard algorithm for the RBM. At present, the contrastive divergence method has become the standard algorithm for the restricted Boltzmann machine. The contrastive divergence method is based on the Gibbs sampling of k steps to obtain $v^{\left(k\right)}$ to approximately obtain the expected value corresponding to the above formula, Gibbs sampling is based on the conditional distribution $p\left(v|h^{\left(k-1\right)}\right)$ to sample $v^{\left(k\right)}$. The update formula for its related parameters are as follows:

(10) $$\Delta w_{ij}\approx P\left(h_{i}=1|v^{\left(0\right)}\right)v_{j}^{\left(0\right)}-P\left(h_{i}=1|v^{\left(k\right)}\right)v_{j}^{\left(k\right)},$$

(11) $$\Delta a_{i}\approx v_{j}^{\left(0\right)}-v_{j}^{\left(k\right)},$$

(12) $$\Delta b_{j}\approx P\left(h_{i}=1|v^{\left(0\right)}\right)-P\left(h_{i}=1|v^{\left(k\right)}\right).$$

The RBM obtains the initial weights and thresholds between the input layer and the hidden layer and between the hidden layers of the deep belief network through such an unsupervised greedy learning method. The initial weights and thresholds between the hidden layer and the output layer of the deep belief network are generated by a random function.

Fine-tuning process: After the pre-training process of the network is completed, the basic structure of the deep belief network can be constructed based on the initial parameters obtained. Then, add another layer of the BP neural network to store the target value of the sample data. The next step is to have a supervised learning of the deep belief network, which is to fine-tune the deep belief network. The fine-tuning process is the same as the conventional neural network parameter optimization process.

However, in addition to selecting a high-performance deep belief network for the large amount of spacecraft telemetry data, efficient algorithms are also needed for the network parameter optimization. The traditional BP algorithm updates the parameter using the gradient descent method, that is, in the opposite direction of the gradient, the parameters are updated in a certain step size to make the loss function reach a minimum value. However, this method ignores the second derivative, and the final phase is linear convergence, and the convergence speed is slow. Therefore, it is suitable for optimizing the initial stage. By contrast, the Newton method uses the second derivative, and the final phase converges quickly and has good convergence, but it is not suitable for the initial phase. Therefore, the gradient descent method is often used in combination with the Newton method. However, the Newton method needs to calculate the Hessian matrix, which is the second derivative. The core idea of the Levenberg–Marquardt algorithm [42] is to replace the Hessian matrix with a Jacobian matrix to improve the algorithm optimization efficiency (Hessian matrix $H=2J^{T}J$, J represents the Jacobian matrix).

The Levenberg–Marquardt algorithm is an improved Newton method and an algorithm for solving the optimal value of a function using non-linear least squares. The algorithm combines the relevant features of the Newton method and the gradient method. When the parameter u is small, the step size is equal to the step size of the Newton method, and when the parameter u is large, the step size is approximately equal to the step size of the gradient descent method. This method is not sensitive to over-parameterization, can effectively deal with the problem of redundant parameters, and reduces the possibility that the function falls into local minimum values. This algorithm can combine the advantages of Newton’s algorithm and the gradient descent method, and improve their insufficiency. The Levenberg–Marquardt algorithm is more advantageous for the optimization of neural network parameters for huge training samples.

Algorithm goal: For function x=f(p), given f(⋅) and the observation vector x containing noise, estimate p.

The specific steps of the algorithm are as follows:

Step 1: Assume that the error precision is constant e, take the initial point $p_{0}$, and calculate the error $e_{0}=\left\|x-f\left(p_{0}\right)\right\|$. Then take k=0, k indicates the number of iterations, b=10 (which can also be any number greater than 1).

Step 2: Calculate the Jacobian matrix $J_{k}$, then calculate $N_{k}=J_{k}{}^{\prime }\cdot J_{k}+u_{k}\cdot I$, I is the identity matrix, $u=10^{-1}$ (u must be greater than 0).

(13) $$J_{k}=\left[\begin{array}{ccc} \frac{\partial f_{1}}{\partial x_{1}} & \cdots & \frac{\partial f_{1}}{\partial x_{n}}\\ \vdots & \ddots & \vdots \\ \frac{\partial f_{m}}{\partial x_{1}} & \cdots & \frac{\partial f_{m}}{\partial x_{n}} \end{array}\right]={\left(\frac{\partial f_{i}\left(x^{k}\right)}{\partial x_{j}}\right)}_{m\times n},$$

Step 3: Construct the corresponding normal equation $N_{k}\cdot d_{k}=J_{k}{}^{\prime }\cdot e_{k}$ and solve for the normal equation to obtain $d_{k}$.

Step 4: If $\left\|x-f\left(p_{k}+d_{k}\right)\right\|<e_{k}$, let $p_{k+1}=p_{k}+d_{k}$, if $\left\|d_{k}\right\|<e$, stop iterating and output the result; otherwise, let $u_{k+1}=u_{k}/b$, and go to step 2.

Step 5: If $\left\|x-f\left(p_{k}+d_{k}\right)\right\|\geq e_{k}$, let $u_{k+1}=u_{k}\cdot b$, re-solve the normal equation to get $d_{k+1}$, and return to step 4.

### 3.3. Characterization of Degradation

A storage battery voltage anomaly detection model is first established and trained based on a DBN. Then, based on the accumulated telemetry parameters of a storage battery, including charge/discharge currents, temperatures and times, the model can be used to continuously predict the charge/discharge voltages of the storage battery. Additionally, the predicted voltages can be compared with the measured voltages of the storage battery. Based on the difference between the predicted and measured voltages, the extent to which the voltage of the storage battery has declined can be determined, thereby realizing real-time diagnosis and provision of warning. Storage battery degradation is classified into four levels, 0, 1, 2 and 3. Level 1 corresponds to slight anomalies, i.e., when the voltage of the storage battery has just started degrading slightly. Level 2 corresponds to moderate anomalies, i.e., when the voltage of the storage battery has degraded significantly. Level 3 corresponds to severe anomalies, i.e., when the voltage of the storage battery has degraded severely. Naturally, level 0 corresponds to when the voltage of the storage battery is at the normal level. The definition of each level is provided as follows.

(14) $$Levels\;of\;abnormality=\left\{ \begin{array}{l} Level\;0,\;if\;{\hat{U}}_{t}-U_{t}<\Delta u\\ Level\;1,\;if\;\Delta u\leq {\hat{U}}_{t}-U_{t}<2\cdot \Delta u\\ Level\;2,\;if\;2\cdot \Delta u\leq {\hat{U}}_{t}-U_{t}<3\cdot \Delta u\\ Level\;3,\;if\;{\hat{U}}_{t}-U_{t}\geq 3\cdot \Delta u \end{array}\right.,$$

where ${\hat{U}}_{t}$ is the voltage predicted by the model, $U_{t}$ is the measured voltage, *t* is time, and Δu is the threshold for voltage degradation (Δu is set to 0.5 V).

## 4. Experimental Validation and Result Analysis

### 4.1. Satellite Background and Data

The experimental data originated mainly from satellite X, as introduced in Section 3.1. Based on its telemetry data, it is found that the satellite X frequently enters and exits the Earth’s shadow region from May through July each year. During this period, the storage battery of the satellite X undergoes notable charge/discharge cycles. Additionally, at the discharge stage, the maximum discharge capacity of the storage battery is low initially but subsequently gradually increases. When approaching the end of the discharge stage, the maximum discharge capacity of the storage battery decreases again. The telemetry data for the storage battery used in this study included the charge currents for the charge controls 1 and 2, the input current for the regulator, the temperatures of the telemetry terminal, the spread spectrum systems A and B and the storage batteries 1 and 2 and the voltage of the storage battery as well as calculated charge/discharge times. The sampling frequency for each of these parameters was once every two seconds. The charge/discharge-stage data screening was performed using the parameter extraction approach described in Section 3.1. Ultimately, storage battery parameter data for the periods of May–July 2014, May–July 2016 and May–June 2016 were obtained. It is worth mentioning that satellite X was still within the planned service cycle in 2014, and its storage battery performed normally. Ground personnel also had not discovered any notable anomalies in the SoH of the storage battery in 2015. However, beginning in May 2016, satellite X started to enter and exit the Earth’s shadow region frequently after being in the sunlit region for a long period of time. The ground personnel noticed a significant decrease in the voltage of the storage battery of satellite X in the discharge mode, so they had to implement some protective measures in June 2016 to prolong the service life of the storage battery. Based on these conditions, this experiment was conducted primarily to determine whether the DBN-based NN model proposed in this study could be used to learn and understand the multivariate correlations between the parameters of the storage battery of satellite X and, on this basis, whether the proposed detection algorithm could be used to discover the anomalies in the SoH of the satellite before and after May 2016, when actual anomalies occurred in satellite X.

The experiment was conducted in a macOS environment on a MacBook Pro with a 3.1 GHz dual-core Intel i5 processor and 8 GB of memory. The code was written and compiled in MATLAB 2016b.

### 4.2. Experimental Results and Analysis

#### 4.2.1. Training effect and Related Comparison of Battery Voltage Anomaly-Detection Model Based on DBN Neural Network Model

This study aims to establish a complex mapping model for multiple storage battery parameters (e.g., charge/discharge currents, times and temperatures) and storage battery voltage for prediction purposes. In order to verify the prediction effect of the DBN neural network model proposed in this paper, this paper chooses to compare with the traditional BP-based neural network model. On this basis, the two parameter optimization methods of gradient descent (GD) and Levenberg–Marquardt (LM) are used to test the prediction performance. The models differed in the selected NN and optimization algorithm but shared the same network structure setting and training targets. Each model contained nine input layers (i.e., the previously introduced parameters, e.g., charge/discharge currents, times and temperatures), 15 hidden layers and 1 output layer (i.e., the voltage of the storage battery). The tahn and purelin functions were selected as activation functions for the hidden and output layers, respectively. Additionally, the maximum number of iterations for NN training was set to 5000.

In the selection of training and test data, the first 70% of the storage battery data for 2014 were used as the training set, and the remaining 30% as the test set to compare the algorithms. On the evaluation criteria for prediction performance, the three evaluation criteria of prediction error accuracy, iteration number and running time are mainly used. Among them, the prediction error adopts the mean square error (*MSE*) and mean absolute error (*MAE*), which were calculated using the following equations:

(15) $$MSE=\frac{1}{n}\sum_{i=1}^{n}{\left(y_{i}-{y^{\prime }}_{i}\right)}^{2},$$

(16) $$MAE=\frac{1}{n}\sum_{i=1}^{n}\left|y_{i}-{y^{\prime }}_{i}\right|,$$

where n is the number of samples, $y_{i}$ is the measured value of the target sample, and ${y^{\prime }}_{i}$ is the predicted value of the target sample. *MAE* was primarily used to calculate the mean loss for the test set. *MSE* was mainly used to enlarge the impact of outliers on the predicted value.

Based on the 2014 data, the model was trained by focusing on the multivariate correlations between the parameters of the storage battery. The DBN was compared with the conventional BP NN. Additionally, the effectiveness of two optimization algorithms, i.e., Levenberg–Marquardt (LM) and gradient descent (GD), was also evaluated. Table 1 summarizes the comparison of the models’ prediction performance. 

As demonstrated in Table 1, the DBM model with the LM optimization algorithm needed only 639 iterations (within the valid number of iterations) to converge. Its operating time was 533.91 s. None of the other models effectively converged within 5,000 iterations. Additionally, their operating time also far exceeded 533.91 s. The MSE (7.25 × 10^−4^) and MAE (0.0105) of the DBN model with the LM optimization algorithm outperformed those of the other models. This suggests that, for the normal dataset, the DBN model with the LM optimization algorithm was able to satisfactorily fit the multivariate correlations between the parameters of the storage battery. Thus, this model was selected as the storage battery voltage prediction model in this study.

**Figure 5:**
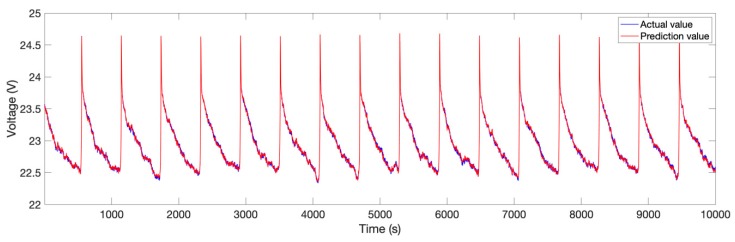
Partial prediction results in 2014 data.

The partial prediction results in 2014 data of the DBN model combined with the Levenberg–Marquardt method are shown in Figure 5. It can be seen that the voltage prediction value fits well with the actual value.

#### 4.2.2. Battery Health Status Online Abnormality-Detection Effect

**Figure 6:**
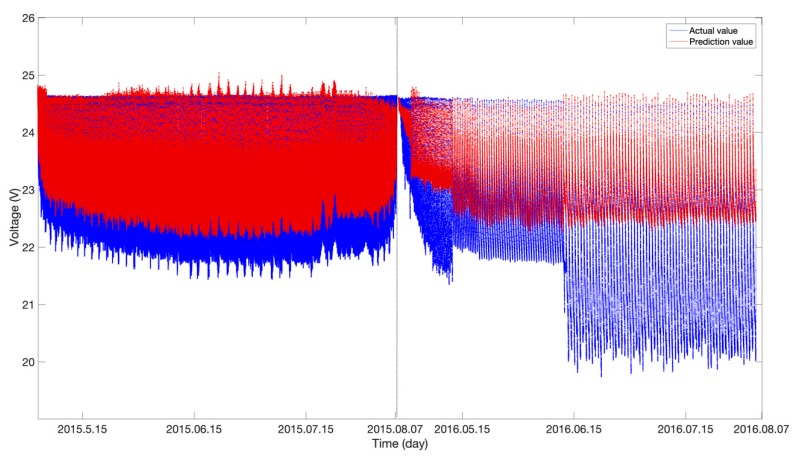
DBN-based comparison of the predicted and measured voltages.

**Figure 7:**
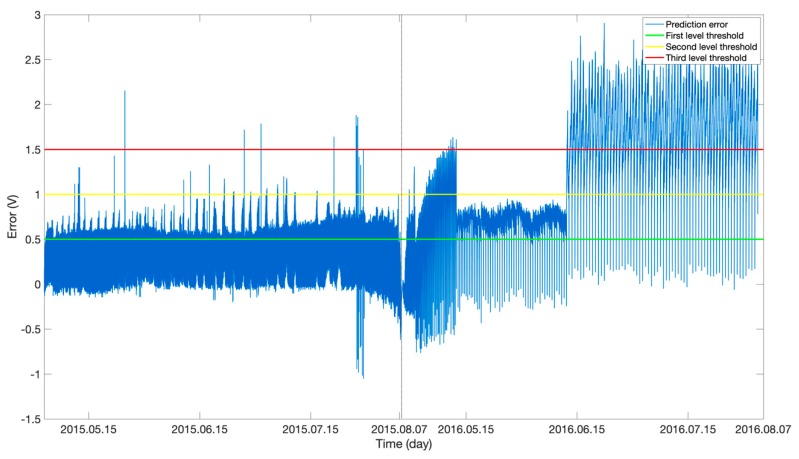
DBN-based prediction errors.

Based on the above DBN NN model trained on the 2014 telemetry data for the storage battery, to further examine whether there were signs of anomalies in the SoH of the storage battery in 2015 and 2016, the trained DBN model was applied to the 2014, 2015 and 2016 test datasets. The error between the measured and predicted values was used to characterize the storage battery degradation. The larger the error was, the more severely the storage battery degraded. Additionally, the long-term degradation trend of the voltage of the storage battery with time was also obtained from long-term observations. Figure 6 shows the predictions obtained (the red and blue curves signify the predicted and measured voltages of the storage battery, respectively). The measured voltage of the storage battery gradually decreased with time. Moreover, the error in the predicted voltage increased gradually. In the final stage when the voltage of the storage battery degraded significantly, the error increased sharply and far exceeded the normal level. Figure 7 shows the prediction error values. As demonstrated in Figure 7, the error values between the predicted and measured voltages for 2014 were basically within the Level-1 anomaly threshold. As the operating time of the spacecraft increased, particularly after it entered the Earth’s shadow region in 2015, the voltage error exceeded the Level-1 threshold, and Level-1 anomalies occurred. In the final stage (i.e., June 2016), the error rapidly increased and exceeded the Level-3 threshold, and severe storage battery degradation occurred. 

**Figure 8:**
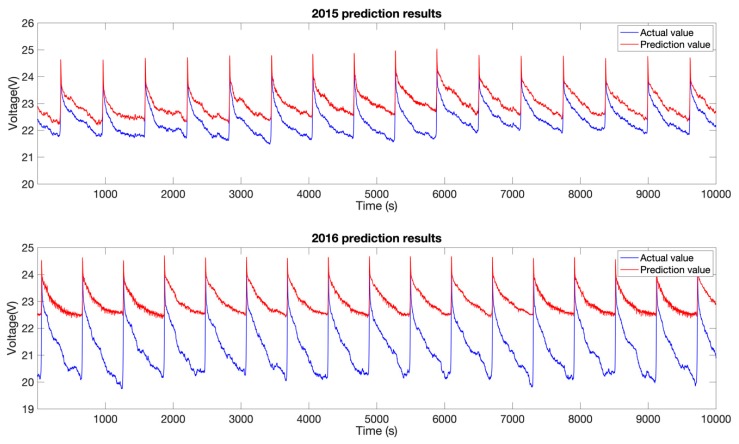
Partial prediction results in 2015 (**above**) and 2016 (**below**).

To visually display the detected anomalies more clearly, the predicted and actual results for 2015 and 2016 are enlarged in Figure 8. The upper figure in Figure 8 shows the predicted voltages in some cycles in the degradation stage in 2015. In this stage, there was a certain difference between the predicted and measured voltages, and the storage battery degraded to a certain extent. The lower figure in Figure 8 shows the predicted voltages in some cycles in the stage when the voltage of the storage battery exhibited significant anomalies in June 2016. In this stage, there was a significant difference between the predicted and measured voltages. The aforementioned results are consistent with the actual conditions described in Section 3.1. In particular, the proposed algorithm was able to detect in a timely fashion the notable anomalies in the SoH of the storage battery of satellite X that occurred in May 2016 after it started to enter and exit the Earth’s shadow region frequently after being in the sunlit region for a long period of time. Additionally, the proposed algorithm was also able to support early detection and retroactive tracking of anomalies. Specifically, while the ground personnel were unable to discover the decline in the SoH of the storage battery of satellite X based on its telemetry parameters observed in 2015, this decline was clearly detected by the proposed detection algorithm. These results suggest that the proposed algorithm is highly effective in the online detection of anomalies in the SoH of storage batteries.

#### 4.2.3. Comparative Verification of Related Multivariate Anomaly-Detection Methods

To examine its effectiveness, the storage battery degradation detection algorithm proposed in this study is compared with two classical multivariate anomaly detection algorithms, namely, PCA and LLE. The detection principle of these two classical algorithms is described as follows. At the offline training stage, normal historical telemetry data Y are first preprocessed and then subjected to dimensionality reduction using a dimensionality reduction algorithm (PCA or LLE). Thus, a low-dimensional spatial coordinate Z is obtained. Based on the original high-dimensional data and the low-dimensional spatial coordinate, a mapping matrix A is calculated. When examining online data, a new test data point $y_{i}$ is first preprocessed, and then its low-dimensional spatial coordinate $z_{i}$ is obtained using the mapping matrix. Afterwards, a reconstructed value ${\hat{y}}_{i}=A^{T}Ay_{i}$ is obtained by reconstruction estimation. The SPE and $T^{2}$ statistics are used to detect anomalies in the low-dimensional coordinate. The control limits for the SPE and $T^{2}$ statistics are calculated based on a certain distribution [20,43]. When the statistics exceed the control limits, it is considered that there are data anomalies; otherwise, the data are considered normal.

The equation for the SPE statistic corresponding to the test data point $y_{i}$ is as follows:

(17) $$SPE_{i}={\left\|{\hat{y}}_{i}-y_{i}\right\|}^{2},$$

The equation for the $T^{2}$ statistic corresponding to the test data point $y_{i}$ is as follows:

(18) $$T_{i}^{2}=z_{i}^{T}\Lambda^{-1}z_{i},$$

where Λ represents the covariance matrix of the low-dimensional embedding coordinate Z of the offline training data, i.e.,

(19) $$\Lambda =\frac{ZZ^{T}}{N-1},$$

**Figure 9:**
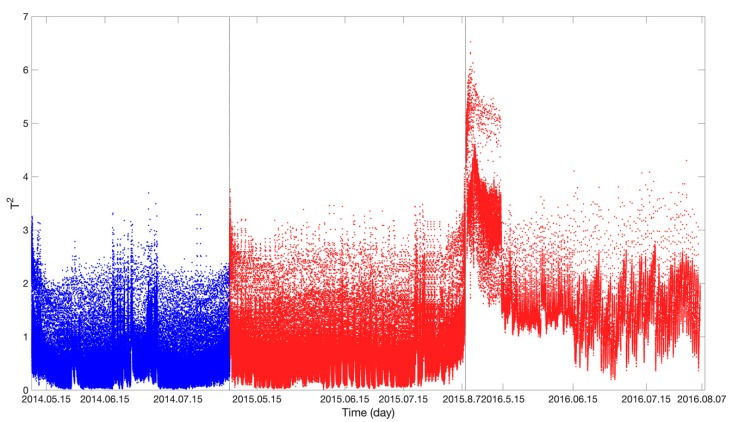
Principal component analysis (PCA) algorithm anomaly-detection result (*SPE*).

Figure 9 shows the results obtained using the PCA anomaly detection algorithm (the blue and red curves show the *SPE* values in the stages when the voltage of the storage battery was normal and degraded, respectively). As demonstrated in Figure 9, there is no significant difference in *SPE* values between the normal and degradation stages. The *SPE* value is only relatively high in the later period of the degradation stage. Anomalies cannot be effectively detected by setting the control limit. In summary, the PCA algorithm is unable to effectively detect voltage degradation when there are large volumes of non-linear storage battery parameter data.

**Figure 10:**
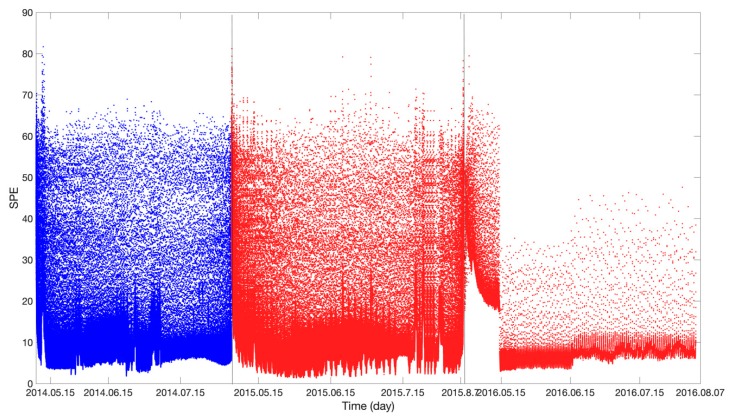
Locally linear embedding (LLE) algorithm anomaly-detection result (*SPE*).

**Figure 11:**
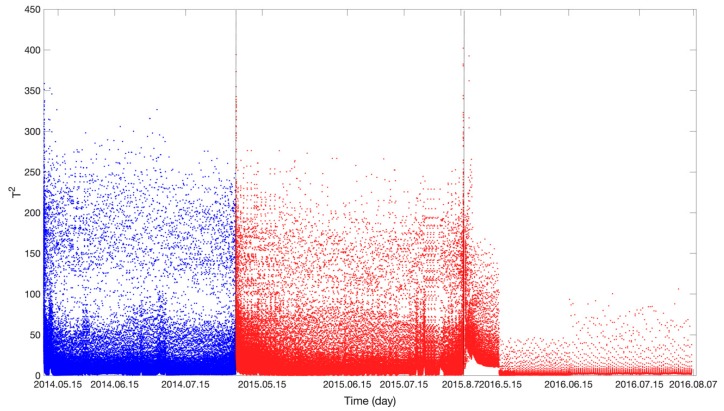
LLE algorithm anomaly-detection result (T^2^).

Figure 10 and Figure 11 show the results obtained using the LLE anomaly detection algorithm. In Figure 10, the blue and red curves show the *SPE* values in the stages when the voltage of the storage battery was normal and degraded, respectively. In Figure 11, the blue and red curves show the *T*^2^ values in the stages when the voltage of the storage battery was normal and degraded, respectively. Similar to Figure 9, there are no significant difference in *SPE* and *T*^2^ values between the normal and degradation stages. The *SPE* and $T^{2}$ values are only relatively high in the later period of the degradation stage. Anomalies cannot be effectively detected by setting the control limit. Therefore, the LLE algorithm cannot effectively detect the voltage decay anomaly compared to the anomaly detection result of the deep belief network.

## 5. Conclusions

Based on the need to detecting anomalies in the SoH of storage batteries of the in-orbit spacecraft as well as the types and characteristics of the accessible telemetry parameters of spacecraft storage batteries, an approach is proposed in this study to characterize the evolutionary pattern of the SoH of a spacecraft storage battery based on the internal correlations between its multiple parameters. Additionally, a DBN model is proposed to learn and train for this evolutionary pattern. On this basis, whether there is an anomaly in the SoH of a spacecraft storage battery is determined based on the difference between the discharge voltage of the storage battery predicted by the DBN model and the actual observed voltage. The proposed DBN model is validated based on the actual telemetry data for the storage battery of a certain in-orbit satellite for a three-year period. It is found that the proposed DBN model can relatively accurately describe the internal correlations between multiple parameters of the storage battery (e.g., the discharge voltage, current, and time, as well as the battery temperature). Furthermore, the proposed DBM model is able to provide the extent of the anomaly in the SoH of the spacecraft storage battery during the period almost one year before the storage battery fault occurred, as well as during the period when the storage battery fault occurred. The proposed DBN model significantly outperforms the conventional BP-based NN model in terms of training time and convergence. The above results show that the proposed DBN model-based voltage anomaly detection algorithm for spacecraft storage batteries is effective and of application value. This algorithm can provide excellent decision-making support for ground personnel to detect anomalies in the SoH of storage batteries of in-orbit spacecraft. Future work will focus on applying the proposed algorithm in the detection of anomalies in the storage batteries of other in-orbit spacecrafts with a view to further modify and improve it.

## Figures and Tables

**Table 1:** Comparison of the prediction performance of neural network (NN) models.

NN Model	Optimization Method	MSE	MAE	Number of Iterations	Time
BP	GD	0.0292	0.1462	5000 (Unconverged)	9472.45 s
BP	LM	0.0013	0.0153	5000 (Unconverged)	4663.37 s
DBN	GD	0.0022	0.0351	5000 (Unconverged)	850.13 s
DBN	LM	7.25 × 10^−4^	0.0105	639	533.91 s
